# Enhancing tidal volume estimation from electrical impedance tomography (EIT) by applying human anthropometric information

**DOI:** 10.1007/s10877-025-01367-y

**Published:** 2025-10-15

**Authors:** Amelie Zitzmann, Anna Strübing, Daniel A. Reuter, Andreas Waldmann, Stephan H. Böhm, Fabian Müller-Graf

**Affiliations:** 1 Department of Anaesthesiology, Intensive Care Medicine and Pain Therapy, University Medical Centre Rostock, Schillingallee 35, 18057 Rostock, Germany; 2 Department of Anaesthesiology, Intensive Care Medicine and Pain Therapy, HANSE-Klinikum Wismar, Störtebekerstraße 6, 23966 Wismar, Germany; 3 EIT Branch, Sentec, Landquart, AG Switzerland

**Keywords:** Electrical impedance tomography, Ventilation, Tidal volume, Respiratory monitoring

## Abstract

**Purpose:**

Electrical impedance tomography (EIT) is a functional imaging technique to monitor regional ventilation. However, the quantification of clinically used ventilation parameters like tidal volume (VT) has not been possible yet since EIT measures relative and not absolute changes in impedance. Thus, the study aimed to evaluate the relationship between impedance changes (dZ) and VT in humans and to identify influencing factors.

**Methods:**

27 patients undergoing elective surgery under general anaesthesia were equipped with a commercially available EIT belt. Measurements were performed at four VTs (6, 8, 10 and 12 mL/BW) on each of four PEEP levels (0, 5, 10 and 15 cmH_2_O). Linear regression analysis was performed for normalized dZ and VT per ideal bodyweight (VT_IBW). Additionally, PEEP, gender, age, height and weight were analysed as potential influencing factors.

**Results:**

Regression analysis for individual patients showed good correlations between VT_IBW and normalized dZ (mean R^2^ 0.890 ± 0.15). However, for the group of patients, correlations were rather weak (R^2^ 0.485). Including additional factors improved the model (adjusted R^2^ 0.767), with VT_IBW having the biggest impact, followed by weight, height and PEEP; age did not contribute to it significantly. Higher VT_IBW, PEEP and height increased, while female gender and higher weight decreased normalized dZ.

**Conclusion:**

Normalized dZ correlated strongly with VT_IBW in the individual ventilated humans but more weakly when analyzing the cohort. PEEP, gender, weight and height were identified as additional influencing factors.

**Trial registration:**

This study was prospectively registered at the German Register of Clinical Studies (Deutsches Register Klinischer Studien; DRKS00027226) on 3rd December 2021.

## Introduction

Electrical impedance tomography (EIT) as a functional imaging technique to monitor the regional distribution of ventilation has been used in human and veterinary medicine [1–6]. As such, it has the potential to become a useful addition to routine clinical monitoring, as it is non-invasive, does not apply ionizing radiation and is available in real-time and right at the bedside [7–9]. Despite these advantages, the quantification of clinically used ventilation parameters like tidal volume (VT) – as repeatedly requested by clinicians – has not been possible so far since EIT measures relative and not absolute changes in impedance. This idea could become helpful not primarily in intubated patients under controlled mechanical ventilation (CMV), where tidal volumes can be measured by the built-in spirometer, but mainly in patients where these measurements fail, e.g. due to high leakage because of broncho-pleural fistula or suboptimal interface fit under non-invasive ventilation; it could even help in estimating VT in spontaneously breathing patients prone to respiratory failure, where declining VTs may precede overt clinical decompensation. Thus, the goal of this study was to evaluate the relationship between ventilation-induced impedance changes (dZ) and the magnitude of VT in humans and to identify additional factors influencing this relationship - primarily in intubated patients under CMV with reliable application and measurement of VT. We hypothesized that a combination of factors which have been suspected to have an influence on the individual VT/dZ-relationships [10] such as age, height and weight, and also different body shapes and thoracic circumferences would allow for estimation of VT. The knowledge gained from this study shall provide the basis for further research into estimating tidal volumes in less controlled situations, such as those mentioned above.

## Methods

### Study design and setting

This single-centre prospective trial was conducted between February and April 2023 at the University Medical Centre Rostock. The study protocol was approved by the ethics committee of the medical faculty of the University of Rostock (file number A 2021 − 0139) and prospectively registered at the German Register for Clinical Studies (DRKS00027226). It was conducted in compliance with the Declaration of Helsinki and ICH Good Clinical Practice (GCP) guidelines.

Adult patients scheduled for elective surgery under general anaesthesia and need for endotracheal intubation were eligible for participation. Further requirements were a minimum duration of surgery of one hour, unrestricted access to the patient’s thorax and no interference with the surgical procedure. Exclusion criteria were pregnancy, body weight (BW) greater than 1.5-fold of ideal body weight calculated using Devine’s formula, ASA physical status class III and above, known lung diseases and contraindications to EIT measurements such as any form of implantable electronic devices, wounds or inflammation at the level of belt placement as well as unstable rib or spinal fractures. Due to the explorative character of the study, sample size calculation was not feasible and the number of patients to be enrolled was set to 30 to gain a representative section of the general population while anticipating for a high drop-out rate.

### Anaesthesia

Conduct of anaesthesia was not influenced by the study protocol and complied with the standard operating procedures of the Department of Anaesthesiology. After induction of anaesthesia, endotracheal intubation was performed with a standard Mallinckrodt or Ring-Adair-Elwyn tube with an inner diameter of 7.0 mm for women and one of 8.0 mm for men. Anaesthesia was conducted as total intravenous anaesthesia (TIVA). Only during data acquisition, responsibility for mechanical ventilation was handed over to the investigators, while overall control of anaesthesia conduct, monitoring and respective interventions remained at the discretion of the anaesthesiologist in charge.

### Study protocol

For EIT measurements, the commercially available single-use EIT belt LuMon Belt™ (EIT-branch, SenTec AG, Landquart, Switzerland) of the appropriate size was placed around the thorax and connected to the LuMon™ monitor (also SenTec AG) according to the manufacturer’s specifications: After assessment of correct size using the measuring tape provided from the manufacturer, the belt was placed with the patient sitting in upright position on the operating table. The top-end of the mid-line marker on the belt’s back was aligned with the processus of the C7 vertebra while the measuring part containing the electrodes of the belt was placed around the chest, just below the arm pits, thus following the 5th intercostal space. To secure the belt’s position the shoulder straps were fastened either around the lateral or the central loops to ensure a symmetrical and snug fit. For subjects with larger breasts, the belt was placed in the inframammary fold to avoid measurement through breast tissue and to ensure stable belt position. After belt placement, signal quality (aim: adequate or strong with no interference) and belt-to-skin contact quality (no failing electrodes) were checked. In addition, a quality check using the ScoutView- and VentView-Screens (breathing-related impedance changes falling mainly within the lung contours, global dynamic images and plethysmogram reflecting patient’s breathing) was performed. For ventilation within the study protocol, an intensive care respirator (Elisa 800, Loewenstein Medical SE & Co. KG, Bad Ems, Germany) was used.

During the protocol, patients were ventilated using volume-controlled ventilation (VCV) at 12 breaths per minute and an inspiration:expiration (I:E) ratio of 1:2. Four PEEP levels (0, 5, 10 and 15 cmH_2_O) were applied in a random order. At each PEEP level, an inspiratory hold manoeuvre was performed to enable synchronisation of the EIT- and ventilator data. Subsequently, four tidal volumes (Vt) of 6, 8, 10 and 12 mL/kg real BW were set in randomized order before changing to the next PEEP level. Each of these 16 conditions was maintained for at least 90 s allowing for equilibration during the first 4–6 breaths before recording data over the following 8–12 breaths. Real body weight was chosen for calculating VT to generate a broader range of different tidal volumes. However, for safety reasons real BW could not exceed the 1.5-fold of IBW (see exclusion criteria); the upper pressure limit for peak airway pressure was set to 30 cmH_2_O.

### Data recording and processing

Ventilatory parameters were recorded at a frequency of 200 Hz by connecting the Scientific Unit (Loewenstein Medical SE & Co. KG, Bad Ems, Germany) to the ventilator. EIT images were acquired at 50 frames per second within the LuMon™ monitor and reconstructed using the modified Graz Reconstruction Algorithm for EIT (GREIT) provided by the monitor. Pseudonymized data were then stored for further analysis using a custom-built MATLAB algorithm (MATLAB™ R2019b, The MathWorks Inc., Natick, Massachusetts USA). Data of the ventilator and the EIT device were matched by using the inspiratory hold manoeuvres performed on each PEEP level. Respiratory cycles within the EIT data were identified by the breath delineation algorithm of the EIT device [11]. The first 4–6 breaths were excluded from analysis (see above) and, after a quality and plausibility check, only the last 8–12 breaths were analysed: for each respiratory cycle, the tidal impedance change (*dZ*) was calculated for the entire tomographic cross section without selecting any region of interest (ROI). For each individual, *dZ* (in arbitrary units) of each breath was normalized by dividing the respective *dZ* by the highest individual *dZ*, with the resulting *dZ_norm* values now ranging from 0 to 1. The corresponding VT (as calculated by the ventilator integrating flow over time) was assigned to this *dZ_norm*. All VTs were also transformed to VT per ideal body weight (*VT_IBW*) as recommended for mechanical ventilation [12]. For each of the 16 conditions mentioned above, mean values of VT_IBW and dZ_norm were calculated per individual.

### Statistical analysis

Data were analysed using Excel (Microsoft, Redmond, Washington, USA) and SPSS Statistics 29 (IBM Corp., Armonk, New York, USA). For each individual, linear regression analysis was performed for *dZ_norm* and *VT_IBW* using the respective mean values. The individual regression coefficients were then analysed with respect to their dispersion and deviation. To evaluate further influencing factors, multiple regression analysis was performed with dZ_norm as dependent variable and VT_IBW, PEEP, gender, age, weight, and height, thoracic circumference and BMI as independent variables. Homoskedasticity was confirmed using a modified Breusch-Pagan test. To exclude multicollinearity, variables with variance inflation factors (VIF) greater than 5 were not analysed. Thus, analysis for BMI and thoracic circumference was not feasible. After identifying all possible combinations of independent variables without multicollinearity, the model with the lowest Akaike information criterion (AIC) was chosen. Mallows’ prediction criterion (Cp) was used to assess model fit and select a parsimonious regression model by balancing goodness-of-fit with model complexity. Shapiro-Wilk tests were used to test for normality of residuals. The root mean square error (RMSE) was used to evaluate the predictive accuracy of the model. P-values below 0.05 were considered significant.

After correction for multicollinearity, multiple regression analysis was performed for:

*dZ_norm =* β_*0*_ *+* β_1_*VT_IBW + β_2_*PEEP + β_3_*gender + β_4_*age + β_5_*weight + β_6_*height.

To account for the factors excluded due to multicollinearity, two-factor regression analysis was performed with: *dZ_norm =* β_*0*_ *+* β_1_*VT_IBW + β_2_*variable.

with *variable * $\in$ *{PEEP, weight*,* height*,* age*,* gender*,* BMI*,* thoracic circumference}.*

Patients’ characteristics are presented as median (interquartile range, IQR), respiratory parameters as mean ± standard deviation (SD) and minimum and maximum. For regression analysis, regression coefficients were used to describe the interpretable effect while standardized coefficients were used to describe the impact of the respective variable on the model; coefficients of determination (R^2^ for linear regression analysis and adjusted R^2^ for multiple regression analysis) are presented to describe the quality of the statistical model.

## Results

Thirty patients were enrolled in the study. While three patients could not complete the protocol as their surgery took place in parallel to others and only one measurement set-up was available at the same time, twelve patients had to be excluded because not all VTs could be applied on all PEEP levels: in most of these cases, at 12 ml/kg real BW the upper pressure limit was reached, and the ventilator did not deliver the expected VT.

Thus, complete data sets of 15 participants could be analysed. Demographic and anthropometric data of the study population are given in Table 1.

**Table 1:** Demographic and anthropometric data of the study population

Parameter	*n* (%)/mean ± SD (min – max)
Gender (m/f)	7/8 (46.6%/53.3%)
Age [years]	44.1 ± 16.8 (22–71)
Real bodyweight [kg]	82.2 ± 19.4 (45–125)
Ideal bodyweight [kg]	64.4 ± 10.2 (50–81)
Height [cm]	171.0 ± 9.2 (157–187)
BMI [m/kg^2^]	27.9 ± 4.8 (18.3–35.7)
Thoracic circumference [cm]	101.2 ± 12.9 (78–120)

*N* = 15; Absolute (relative) frequencies are given for gender. All other data are given as mean ± standard deviation (SD) together with minima and maxima (min – max)

Actual tidal volumes, tidal volumes per real body weight and per ideal body weight are presented in Table 2.

**Table 2:** Applied tidal volumes

Intended VT [ml/kg]	VT as calculated by ventilator [mL]	VT per real BW [mL/kg]	VT per ideal BW [mL/kg]
6	493.0 ± 113.7 (267.1–760.7)	6.0 ± 0.08 (5.8–6.2)	7.6 ± 1.1 (5.3–9.4)
8	655.9 ± 152.6 (356.7–1000.0)	8.0 ± 0.07 (7.8–8.2)	10.1 ± 1.6 (7.1–12.3)
10	822.8 ± 186.5 (454.3–1247.3)	10.0 ± 0.1 (9.7–10.2)	12.8 ± 2.0 (9.1–15.4)
12	982.3 ± 225.0 (534.1–1494.8)	12.0 ± 0.2 (11.5–12.4)	15.2 ± 2.3 (10.7–18.5)

Mean tidal volumes ± standard deviation (minimum - maximum). Tidal volumes are expiratory volumes. Leakage < < 1%

Highest dZ values were always recorded at the highest VT but were evenly distributed over the PEEP levels (3x at PEEP 0 cmH_2_O, 4x at 5 cmH_2_O, 5x at 10 cmH_2_O, and 3x at 15 cmH_2_O). No significant impact of demographic data on these values was seen.

For the individual patient, regression analysis showed a good correlation between dZ_norm and VT_IBW (mean R^2^ 0.89 ± 0.15; 0.40–0.99) with regression coefficients (β) between 0.05 and 0.07 (mean β 0.06 ± 0.01). For the group of patients, R^2^ was 0.49 and β was 0.04. After excluding one outlier with bad intra-subject correlation, R^2^ was 0.59, β remained 0.04.

**Fig. 1:**
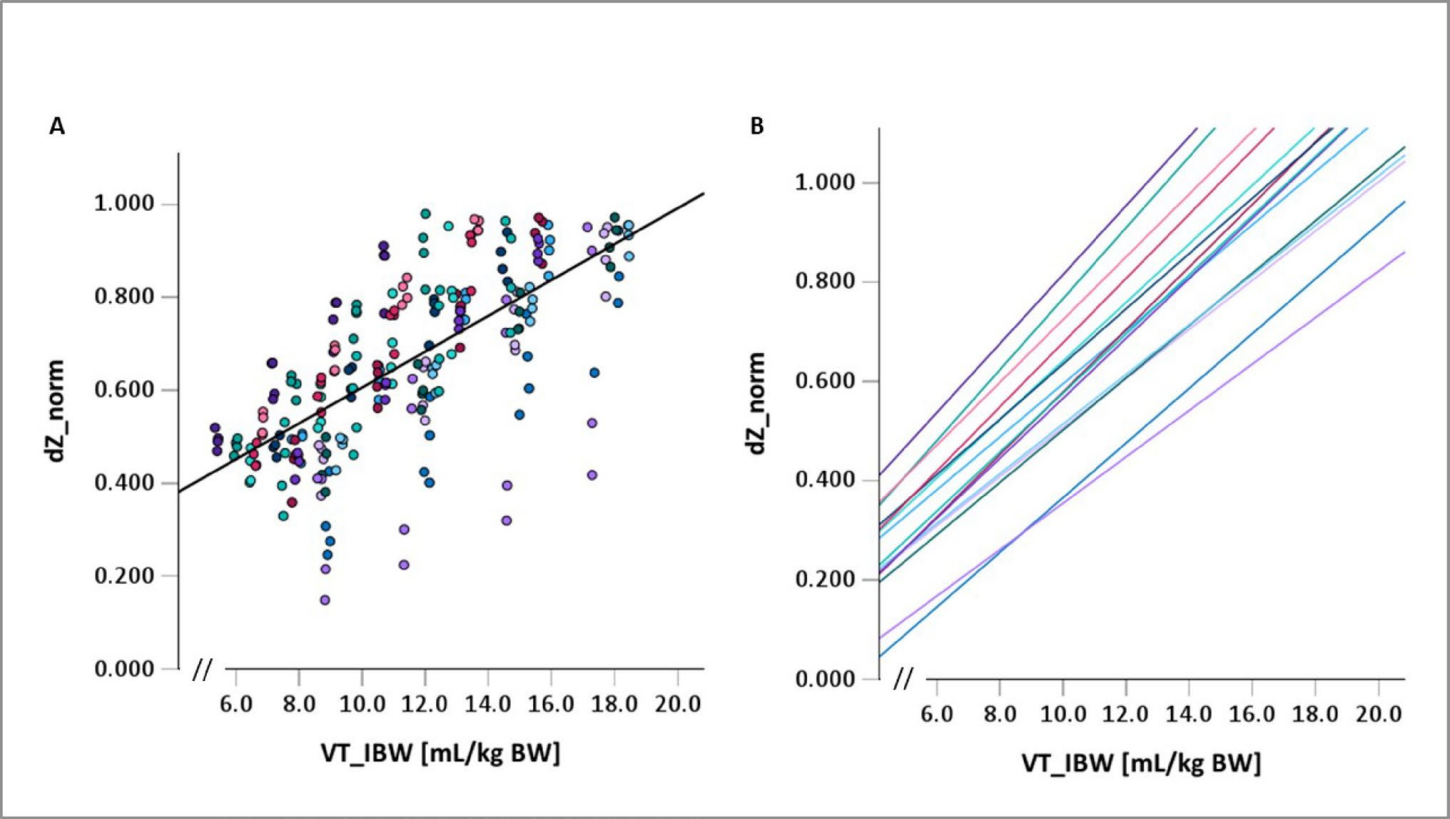
Regression analysis **A **DZ_norm at the respective VT_IBW with the overall regression plot (bold black line; R2 = 0.49; β = 0.04). **B**: The respective regression lines for each of the 15 individuals (colour coded) with mean R2 0.89 and mean β 0.06. Please note that the x-axis is not shown from the origin

After multicollinearity analysis, the model with *dZ_norm =* β_*0*_ *+* β _1_*VT_IBW + β _2_*PEEP + β _3_*gender + β _4_*age + β _5_*weight + β _6_*height had the lowest AIC and was chosen. Multiple regression analysis with VT_IBW, PEEP, gender, age, height and weight as independent variables had an adjusted R^2^ of 0.77 with VT_IBW, PEEP, gender, height and weight as significant contributors (*p* < 0.01), while age did not contribute significantly (*p* = 0.599). The respective regression coefficients and standardized coefficients are listed in Table 3. The RMSE for a model including all patients was 0.09; Cp was 7. Omitting the outlier, adjusted R^2^ was 0.854 and the RMSE for this model was 0.07.

**Table 3:** Multiple regression analysis

independent variable	regression coefficient (β)	standardized coefficient
VT_IBW	0.055	0.998
height	0.013	−0.974
weight	−0.010	0.629
PEEP	0.004	0.108
gender	−0.083	n.a.

Multiple regression analysis for dZ_norm as dependent variable with VT_IBW, PEEP, gender, height, weight and age. Only parameters with significant regression coefficients are listed. While regression coefficients describe the functional relationship between the independent variable and dZ_norm, standardized coefficients represent the impact of the respective parameter (n.a.: not applicable for binary variables such as gender)

With the help of the following regression equation, dZ_norm – in our study – could be predicted from VT_IBW, PEEP, height, weight, and gender (*male = 0; female = 1*):

$$\begin{aligned}\:dZ\_norm=&\:-1.412+0.055*V{T}_{IBW}+0.004*PEEP-0.083*gender+0.013*height-0.010*weight\end{aligned}$$

Thus, gender-specific tidal volume can be predicted for the group by the following formulas:

$$\begin{aligned}&\:V{T}_{IBW}\:\left(female\right)=\frac{dZ\_norm+1.495-0.004*PEEP-0.013*height+0.010*weight}{0.055}\end{aligned}$$

$$\begin{aligned}&\:V{T}_{IBW}\:\left(male\right)=\frac{dZ\_norm+1.412-0.004*PEEP-0.013*height+0.010*weight}{0.055}\end{aligned}$$

BMI and thorax circumference were excluded from the above-mentioned multiple regression analysis due to multicollinearity; thus two-parameter regression analysis was performed. The adjusted R^2^ values for dZ_norm vs. VT_IBW and the parameters were: BMI (0.63), weight (0.55), thorax circumference (0.53), height (0.49), age (0.49), PEEP (0.49) and gender (0.48).

The impact of the different PEEP levels is given in Table 4. Mean R^2^ for the individual regression analysis was 0.997 ± 0.003 (0.986–1.000).

**Table 4:** Regression coefficients and coefficients of determination (R^2^) values at the respective PEEP levels

	Regression coefficient (β)	Coefficient of determination (*R*^2^)
PEEP 0 cmH_2_O	0.061 ± 0.01 (0.031–0.081)	0.997 ± 0.003 (0.989–1.000)
PEEP 5 cmH_2_O	0.061 ± 0.01 (0.036–0.077)	0.997 ± 0.004 (0.986–1.000)
PEEP 10 cmH_2_O	0.058 ± 0.01 (0.051–0.068)	0.998 ± 0.001 (0.996–1.000)
PEEP 15 cmH_2_O	0.054 ± 0.01 (0.040–0.068)	0.997 ± 0.003 (0.991–1.000)

Data presented as mean ± standard deviation (minima – maxima)

**Fig. 2:**
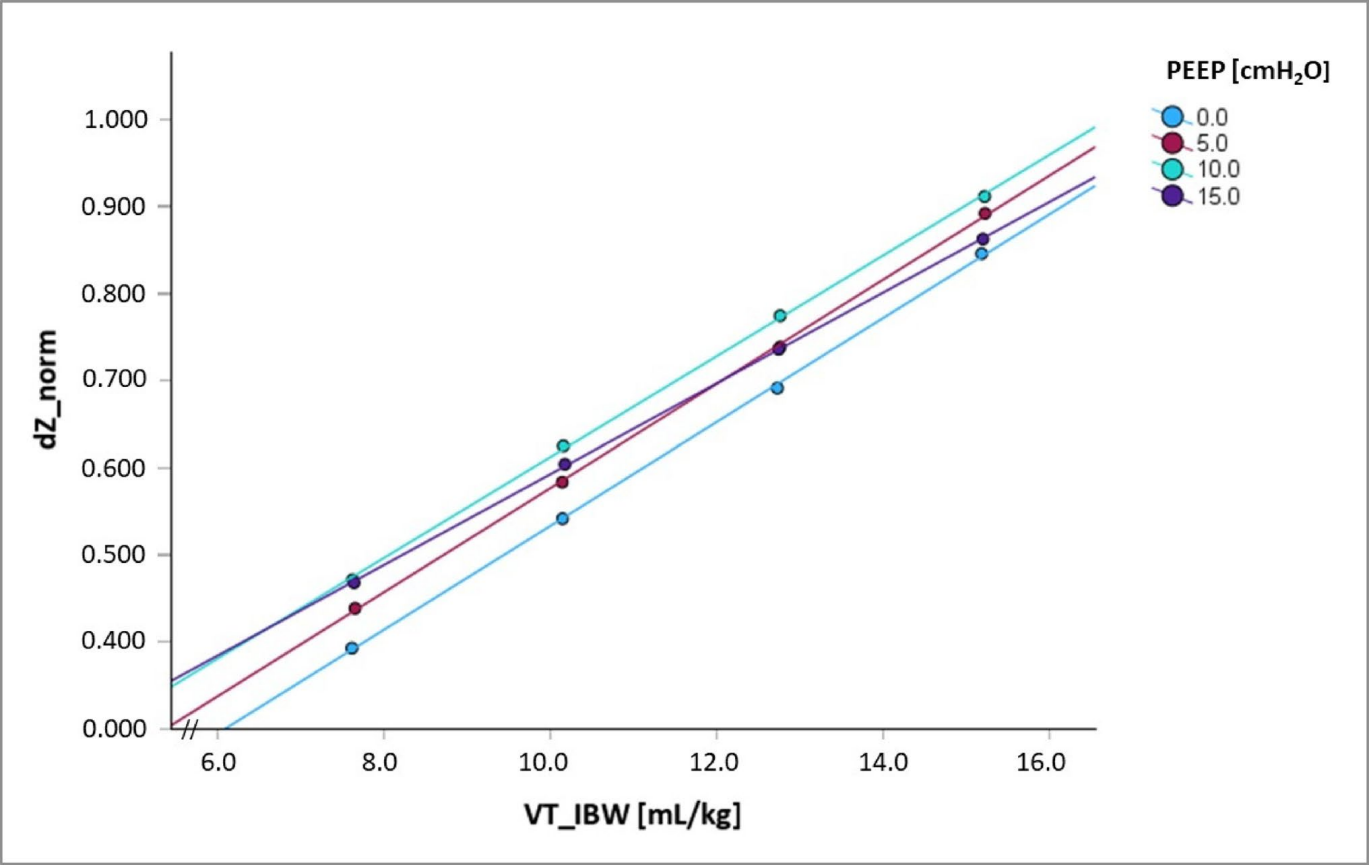
Regression analysis at the respective PEEP levels. Regression analysis for dZ_norm of all patients as dependent variable with VT_IBW for the four different PEEP levels. While regression lines for PEEP 0 cmH_2_O, 5 cmH_2_O and 10 cmH_2_O run in parallel, the one for PEEP 15 cmH_2_O is significantly flatter, crossing the other three lines

Regression lines for the group of patients at the four PEEP levels are presented in Fig. 2. Related-samples Friedman’s Two-Way ANOVA by ranks showed significantly lower regression coefficients at PEEP 15 cmH_2_O than at PEEP 0 cmH_2_O (*p* < 0.01), while coefficients at the other levels did not differ significantly from each other.

## Discussion

Regression analysis in our cohort revealed a linear correlation between normalized impedance changes (dZ_norm) and the respective tidal volumes (VT_IBW), which confirms observations in humans [10, 13] and other species like horses [14, 15], cattle [7], and pigs [16]. However, only 49% of the variance of dZ_norm during changes of VT could be explained by VT_IBW alone; even when omitting one patient with markedly worse correlations than the other subjects, this value rose to only 59%. Nevertheless, these values are lower than in previous human studies [10, 13, 17]. These correlations could, however, be improved by adding further patient- and ventilation-specific factors: multiple regression analysis with PEEP, height, weight and gender could now explain 77% of the variance of dZ_norm when increasing VT_IBW. In addition to VT_IBW, weight and height had a particular influence on the changes in dZ_norm, while PEEP had only a minor impact (as expressed by the standardized coefficients). Female gender and higher body weight lead to a lesser increase in dZ_norm when VT_IBW was increased. Greater height and higher PEEP levels (the latter at least up to certain limits; see below), lead to higher increases in dZ_norm per unit VT_IBW. Age did not play a significant role in the dZ_norm/VT-relationship. Thus, in our study, adding the abovementioned factors accounted for 28% of variance in the dZ_norm/VT_IBW correlation, which is in contrast to the results found by Marquis and colleagues [10], who reported only 1.3% variance in a comparable collective of spontaneously breathing humans. This difference might be due to alternative belt positioning: the Canadian group placed their belt further caudal at the level of the 10th thoracic vertebra, which can significantly impact ventilation measurements, particularly the dZ/VT ratio [18–20]. How EIT signals during controlled mechanical ventilation differ from those in spontaneously breathing subjects should be subject of further investigation.

As seen in previous studies [10, 14, 15, 17], analysis of the individual dZ_norm together with the respective VT_IBW showed good linear correlation for most of our patients (mean R^2^ 0.89) with only one outlier. Inter-subject differences in the slope of the regression lines between 0.05 and 0.07 were noted. From the results of our study, estimation of VT by EIT from dZ seems possible using height, weight and gender as additional correction factors in intubated and mechanically ventilated patients. With a broad span in age, height and especially weight – together with a wide range of tidal volumes on different PEEP levels, this study covers many aspects of the clinical spectrum. Nevertheless, regression factors (βs) should be evaluated and redetermined in a larger cohort, as this small exploratory study can only serve to prove the concept, but analysed too few patients to provide generalisable numerical values. The logical next steps in our research agenda would be testing the concept in intubated patients under (assisted) spontaneous breathing and in patients under non-invasive ventilation before applying the concept to spontaneously breathing patients without mechanical support.

An individualized, subject-specific (two point) calibration, e.g. with spirometry, during either controlled or assisted ventilation could provide more accurate results [16, 17, 21]. Further research is needed to evaluate for how long such a calibration would be valid as factors such as drift and skin resistance also play a role in the generation of dZ. When this kind of calibration is not possible, e.g. during non-invasive ventilation with non-tight facial interfaces or spontaneous breathing where the estimation and trending of VT are of particular importance, the abovementioned factors like height, weight and gender can help in estimating VT from dZ more reliably.

As PEEP is the only factor adjustable by the physician in charge, we decided to analyse the data obtained on the different PEEP levels separately. Frerichs and colleagues showed a PEEP-dependency of dZ in pigs: the higher the PEEP the higher the end-expiratory dZ [13]. This can also be seen in the regression curves of the means from our study: for PEEP 0, 5 and 10 cmH_2_O the curves run almost parallel with higher PEEP levels resulting also in higher dZ. At PEEP 15 cmH_2_O, however, the slope of the regression line became flatter meaning that the effect of this higher PEEP became weaker as tidal volumes increased. One explanation might be a PEEP-induced overinflation. Thus, when adjusting for the level of PEEP used, the individualized correlations became even higher. With higher PEEP, higher amounts of air remain within the lungs at the end of expiration (end-expiratory lung volume = EELV) such that the air from the subsequent tidal volume is distributed differently within the lungs than at lower PEEP levels with less or no overdistension [22]. Furthermore, discussions are ongoing as to whether the tidal breathing-induced impedance change dZ represents mechanical strain rather than tidal volume [23], where for mechanical ventilation strain is defined as the ratio of change in lung volume (i.e. VT) to the resting lung volume, i.e. EELV [24]. For our study this would imply that for a given tidal volume delivered at a lower PEEP the ratio of VT/EELV and thus the assumed strain must increase. However, our data did not show an increase in dZ_norm with lower PEEPs. On the contrary, higher PEEP led to higher dZ_norm for PEEP levels 0 to 10 cmH_2_O. Therefore, the association of dZ with strain is rejected.

### Clinical application and prospects

In ventilated patients, tidal volume is typically measured via the ventilator using flow-based spirometry. Therefore, an additional measurement using EIT does not initially seem necessary. However, the working group’s aim is to enable tidal volume to be determined independently of the type of ventilation. This includes situations in which spirometry becomes inaccurate, for example in cases of high leakage due to bronchopleural fistulas during controlled ventilation, or in cases of poorly fitting interfaces during non-invasive ventilation. In such cases, dZ represents true pulmonary ventilation, while spirometric data become irrelevant. Conversely, EIT-based measurement could be used in clinical situations where tidal volume is not routinely measured, such as in patients who are spontaneously breathing and only receiving oxygen therapy. For these patients, a tidal volume that is decreasing to the point where it can no longer be compensated for by an increase in respiratory rate is often the first sign of impending respiratory failure, even before this becomes apparent in pulse oximetry or blood gas analysis. Nevertheless, for these measurements, the method must first be validated under controlled conditions and confounding factors must be identified. These controlled conditions can only be achieved by controlled ventilation under general anaesthesia, which also enables reliable determination of tidal volume using a validated measurement method via spirometry inside the ventilator. Therefore, the present study design was used for this initial approach to the topic. We acknowledge that some of the factors employed in the multifactorial analysis may become less relevant in different contexts. One example is PEEP, which we have included in the model as an important component that has become indispensable in modern ventilation medicine and has been shown to influence dZ. However, PEEP might become less relevant for an analysis of spontaneously breathing subjects, and the resulting model must be adjusted accordingly.

This analysis could provide an equation for calculating VT from dZ_norm in combination with PEEP, gender, height and weight for mechanically ventilated patients. However, intraindividual correlations were higher, thus suggesting personalised approaches whenever possible.

### Limitations

Out of 30 patients that were included in the study, 3 could not undergo the protocol due to organisational reasons. Of the remaining 27 patients, only 15 patients had measurements at all tidal volumes and PEEP levels and thus, underwent analysis. The main reason for the missing measurements was that at 12 ml/kg real BW the upper pressure limit was reached, and the ventilator did not deliver the expected VT. We decided deliberately to relate the tidal volumes to the real body weight for the protocol to achieve a wider spread of the data. For the subsequent analysis, however, the tidal volumes were then related to the ideal weight as usual [12]. With this approach, we achieved a wide span of VTs, but on the other hand, had to accept that upper safety limits for ventilation were reached.

Our study was conducted in subjects without known lung disease that had to undergo endotracheal intubation for the purpose of elective surgery under general anaesthesia. Studies investigating the effects of PEEP and VT using EIT have mostly been performed in humans with acute respiratory distress syndrome (ARDS) or in animal models and might therefore lack comparability [14, 15, 25].

There was one patient with markedly worse individual correlations (R^2^ 0.390). The subject was a small (160 cm), slightly obese (BMI 29.3 kg/m^2^), 35-year-old female with breast implants. During the measurement no deviations or alterations of the EIT signals were noticed. Nevertheless, unnoticed incorrect belt positioning could result in poor agreement in the measurements, as described before [16]. From our own experience this is especially true for small obese females, where diaphragmatic, colonic and gastric movements within the EIT belt plane have caused out of phase signals (unpublished). The same effects have also been described and confirmed by CT scans for spontaneously breathing and mechanically ventilated horses [1, 2]. Furthermore, especially at lower tidal volumes cardiac-related impedance changes could interfere with the measurements [16]. As the reason for these correlations being worse than in other subjects remains unclear, we stated results with and without data of this patient.

## Conclusion

Individualized regression analysis found stronger correlation than the analysis performed for the entire group with a wide distribution of demographic and anthropomorphic factors. During multiple regression analysis, PEEP, gender, weight and height contributed to better correlations between dZ_norm and VT, whereas age had no significant impact.

## Data Availability

Source data can be obtained from the authors upon reasonable request.
